# Efficacy of Insulin Titration Driven by SMS in Improving Glycemic Control in People with Type 2 Diabetes

**DOI:** 10.3390/jcm12196364

**Published:** 2023-10-04

**Authors:** Ángel Ortiz-Zúñiga, Olga Simó-Servat, Judit Amigó, Mónica Sánchez, Carla Morer, Josep Franch-Nadal, Regina Mayor, Tim Snel, Rafael Simó, Cristina Hernández

**Affiliations:** 1 Endocrinology and Nutrition Department, Vall d’Hebron Hospital Campus, 08035 Barcelona, Spain; olga.simo@vallhebron.cat (O.S.-S.); judit.amigo@vallhebron.cat (J.A.); monica.sanchez@vallhebron.cat (M.S.); rafael.simo@vhir.org (R.S.); 2 Diabetes and Metabolism Research Unit, Vall d’Hebron Research Institute, 08035 Barcelona, Spain; 3 CIBER de Diabetes y Enfermedades Asociadas (CIBERDEM), Instituto de Salud Carlos III, 28029 Madrid, Spain; josep.franch@gmail.com; 4 Primary Health Care Center EAP 8K Rio de Janeiro, Institut Català de la Salut, UTAC Muntanya, 08016 Barcelona, Spain; cmorer.bcn.ics@gencat.cat; 5 Primary Health Care Center Raval Sud, Gerència d’Atenció Primaria, Institut Català de la Salut, 08001 Barcelona, Spain; 6 Roche Diabetes Care Spain SL, 08174 Barcelona, Spain; regina.mayor@roche.com; 7 Roche Diabetes Care Nederland B.V., NL-1322 Almere, The Netherlands; tim.snel@roche.com

**Keywords:** type 2 diabetes, insulin treatment, clinical care, telemedicine, text messaging

## Abstract

Aim: To evaluate the efficacy of the self-management of insulin titration based on information received by the Short Message Service (SMS). Methods: A case-control study including 59 subjects in each arm with 16 weeks of follow-up was performed. The inclusion criteria were: (1) Subjects with type 2 diabetes (T2D) under basal insulin treatment; (2) Suboptimal glycemic control: HbA1c ≥ 7.5% and fasting capillary blood glucose (FCBG) > 140 mg/dL (>3 times per week). Subjects were invited to use an insulin titration service based on SMS feedback aimed at optimizing glycemic control depending on fasting blood glucose levels. Psychological aspects were evaluated in the interventional group by means of validated questionnaires (DDS, HADS and SF-12). Results: The intervention group achieved a lower mean FCBG (126 mg/dL ± 34 vs. 149 mg/dL ± 46, *p* = 0.001) and lower HbA1c (7.5% ± 1.3 vs. 7.9% ± 0.9, *p* = 0.021) than the control group. In addition, the intervention group showed a significant improvement in psychological aspects related to Emotional Burden (*p* = 0.031), Regimen Distress (*p* < 0.001), Depression (*p* = 0.049) and Mental Health (*p* < 0.01). Conclusions: The SMS-guided titration was effective in terms of improving glucometric parameters in comparison with the standard of care and improved significant psychological aspects—mainly, the stress associated with insulin treatment

## 1. Introduction

The importance of glycemic control in preventing and delaying the progression of diabetic complications is well established [1,2]. Diabetes management guidelines typically advocate a target glycated hemoglobin (HbA1c) value of 6.5 or 7.0%. However, glycemic management must be individualized considering a less tight control (HbA1c between 7% and 8%) for subjects with severe hypoglycemia risk, those who are elderly, those with a limited life expectancy and those with extensive comorbid conditions [3].

Type 2 diabetes (T2D) is the most common and clinically important metabolic disorder, which has become a global pandemic in recent decades and a major healthcare burden worldwide. Whereas insulin insensitivity is an early phenomenon partly related to obesity, pancreas beta-cell function declines gradually over time already before the onset of clinical hyperglycemia [4]. Despite the availability of a range of antidiabetic therapies, many people with type 2 diabetes (T2D) require insulin treatment at some stage in their disease management. Thus, insulin should be considered for people with T2D when they become insulin-deficient or when non-insulin antidiabetic therapies fail to achieve the target glycemic control [3]. It is well known that a timely and appropriate insulin replacement therapy prevents diabetic complications and therefore reduces healthcare expenses [2].

An important aspect related to the difficulty of achieving and maintaining optimal glycemic control is clinical inertia, defined as the failure to initiate or intensify therapy when required [5,6,7,8,9,10]. Clinical inertia for insulin has been extensively studied in people with T2D [5,6,7,11,12,13,14], and it is well documented in western countries. After the initiation of insulin treatment, the glycemic control improves significantly. The success of insulin therapy relies on the associated titration schedule aimed at achieving the target objectives.

The onset with insulin therapy is a challenge for people, healthcare professionals and the healthcare system. The causes of the delay in adjusting insulin titration are complex and include concerns from subjects, such as fear of hypoglycemia and weight gain. This stressful situation is frequently associated with a reduction in even missed doses of insulin, which preclude obtaining good glycemic control [15,16,17]. A key point in overcoming clinical inertia is to schedule frequent appointments for adjusting insulin treatment. However, in clinical practice, insulin adjustments are carried out in wide intervals during outpatient clinic visits (up 6 months in many cases), being in some cases insufficient to cope with all the doubts and difficulties that people may encounter during this process. In addition, the increase in T2D prevalence makes the capacity of healthcare providers (HCP) insufficient in meeting this need.

In this scenario, feasible methods of insulin titration for achieving glycemic targets without increasing the burden on the healthcare system become very relevant. On this basis, the aim of the present clinical study was to evaluate the efficacy of an SMS service in improving basal insulin titration for subjects with T2D with suboptimal glycemic control.

## 2. Materials and Methods

### 2.1. Design, Subjects and Inclusion Criteria

A 16-week clinical trial follow-up was performed. The study population included subjects with T2D and suboptimal glycemic control who attended primary healthcare clinics in the engagement area of Vall d’Hebron University Hospital (Barcelona Nord).

The Ethic Committee of the Vall d’Hebron University Hospital approved all the procedures PR(AG)506/2020.

The study participants were divided into two groups:

− Cases (intervention group): subjects with T2D that used an SMS-based insulin titration service. Cases were recruited from Primary Health Care Centers (PHCC) of our engagement area.
− Controls: subjects with T2D under the standard of care. This group was obtained from electronic records belonging to the System for the Development of Research in Primary Care (SIDIAP) database. This comprises the clinical information coded in the corresponding medical records from the PHCC of Institut Català de la Salut (ICS). The control group was matched with each case (1:1) by age (±3 years), sex, diabetes duration (±3 years), body mass index (BMI) (±3), diabetic complications and socioeconomic and geographical area (from the same PHCC as the cases).

The inclusion criteria were: (a) Signed, written, informed consent for the interventional group; (b) Males and females with at least 35 years of age; (c) T2D under basal insulin treatment (evening or bedtime) with glargine for at least 6 months but less than 5 years; (d) Suboptimal glycemic control: HbA1c ≥ 7.5% (58.5 mmol/mol), determined within the previous 3 months, and/or fasting capillary blood glucose (FCBG) >140 mg/dL (more than three times the week before the study entry); (e) Possessing and using a mobile phone including messaging and having access to the mobile phone network at home.

The exclusion criteria were: (a) Subjects with HbA1C > 9.5% or using more than 50 U/day of basal insulin; (b) Body max index (BMI) < 25 Kg/m^2^; (c) Subjects in whom other hypoglycemic treatments were added after starting the study. These criteria were included to avoid drop-outs due to the requirement of rapid-acting insulin in this subset of the population.

### 2.2. Methods

The optimization algorithm was proposed by the health personnel of the study in an individualized way according to the weight, insulin sensitivity factor and FCBG level of the participants. Once the healthcare professional had activated the service within the Accu-Chek Smart Pix software 3.2.5 and defined the titration rules for the subject, the titration service started. Via text message (SMS), the service provided, every three days, a recommendation on how to increase the insulin dosage to adjust the basal insulin dose until the blood glucose levels were within the target range (70–139 mg/dL). This optimization algorithm was based on the weight, insulin sensitivity factor and FCBG level of the participants. We defined stable FCBG as when fasting capillary glucose levels were between 70 and 139 mg/dL for 3 consecutive days.

The RocheDiabetes InsulinStart service sends two SMS messages to the participants every day—one in the morning and one in the evening. The SMS message in the morning reminds the participants to measure their blood glucose level before breakfast. The SMS message in the evening recommends the amount of insulin the participants should inject based on the blood glucose levels and insulin information shared by the participants via SMS. The RocheDiabetes InsulinStart service ends automatically once the blood glucose results are stable within the target range for 3 consecutive days. At this point, the participants receive a completion message giving guidance on how to continue. The Accu-Chek Smart Pix software provides a report on the insulin adjustment history to the healthcare professionals.

To evaluate psychological issues, we used validated questionnaires such as change in Diabetes Distress Scale (DDS) [18], Hospital Anxiety and Depression Scale (HADS) [19] and Social Functioning (SF-12) [20] in the intervention group at the baseline and the end of the study (16-week follow-up). In addition, to evaluate the satisfaction regarding the use of an SMS-based insulin titration service, we developed a specific questionnaire.

The DDS is a measure of diabetes-related distress [18]. It consists of 17 items scored on a 1–6 scale, with higher scores indicating higher distress. The DDS comprises four subscales (emotional burden, physician-related distress, regimen-related distress and interpersonal distress) and a total score. A total or subscale score >2.0 is considered clinically significant [21].

The HADS comprises 14 questions: 7 specific to anxiety and 7 specific to depression. A four-point category scale (0–3) is used to measure participants’ responses, with a total score of 21 possible for both the anxiety and depression scales. Scores between 0 and 11 are considered normal, and scores higher than 11 suggest the presence of a possible clinical level of anxiety or depression requiring further assessment.

To assess the quality of life (QoL), the 12-item Short-Form Health Survey-Version 2 (SF-12v2) scale was used. It was designed to assess the multidimensional elements of physical and mental health in the general population as well as people suffering from chronic conditions.

The study schedule is shown in Supplementary Table S1.

### 2.3. Primary and Secondary Endpoints or Objectives

The primary endpoint was the improvement of glucometric parameters (mean FCBG at the last week and HbA1c at the end of the study). The secondary endpoint was the number of severe hypoglycemic events.

In the intervention group (cases), additional secondary endpoints were evaluated: (a) Number of weeks until the FCBG target range was reached; (b) Change in the post-prandial glucose measures; (c) Daily basal insulin dose required after reaching the FCBG target range; (d) Change in the Diabetes Distress Scale (DDS) [18]; (e) Change in the Hospital Anxiety and Depression Scale (HADS) [19]; (f) Change in Social Functioning (SF-12) [20]; (g) Number of hypoglycemic events, i.e., self-monitored BG < 70 mg/dL (3.9 mmol/L); (h) Subject satisfaction regarding the use of an SMS-based insulin titration service; (i) Adherence to the requests of an SMS-based insulin titration service; (j) Number of contacts with the HCP and other study staff concerning the use of an SMS-based insulin titration service.

Poor adherence was defined as reaching <80% compliance for the SMS response during the follow-up.

### 2.4. Statistical Analysis

The sample size was calculated considering that 30% of subjects under the standard-of-care treatment would accomplish the FCBG target and assuming that, in the intervention arm, this effect would increase to 55%. Considering an alpha error level of 5%, the one-tailed calculation gave a power of over 80%, with 47 participants in each of the control and intervention groups. Assuming a drop-out of 20% during the follow-up, a total of 59 participants were required in each group.

Primary analyses were conducted per protocol (restricted to participants who completed the study). A descriptive analysis was carried out for each variable investigated. Frequencies were calculated for qualitative variables, and the mean, standard deviation and interquartile range were calculated for quantitative variables. The normal distribution of the variables was evaluated using the Kolmogorov–Smirnov Test. All the variables analyzed followed a normal distribution. Comparisons between the groups were performed using the Student’s *t* test for continuous variables and the chi-squared test for categorical variables. To evaluate the intragroup differences during the follow-up, paired *t*-tests were used. Finally, we performed a multivariate analysis to determine the independent predictors of poor adherence to the SMS service.

## 3. Results

The subject’s baseline characteristics are shown in Table 1.

A total of 51 subjects completed the study (drop-out: 13.6%). The mean FCBG (126.1 mg/dL ± 34.1 vs. 149.2 mg/dL ± 46.2, *p* = 0.001) and the HbA1c (7.5 ± 1.3 vs. 7.9 ± 0.99, *p* = 0.008) at 16 weeks of follow-up were lower in the interventional group than in the controls. The evolution of glucometric parameters in subjects that completed the study is shown in Table 2. The percentage of subjects who achieved a stable FCBG (between 70 and 139 mg/dL) at 16 weeks of follow-up in the intervention group using an SMS insulin titration service was higher than that in controls (59.5 ± 4.4% vs. 42.4 ± 4.4%; *p* = 0.04). In the intervention group, the time needed to achieve a stable FCBG was 5.1 ± 3.9 weeks.

The final basal insulin dose in the intervention group was 0.45 ± 0.3 Ui/Kg/day vs. a final basal insulin dose of 0.39 ± 0.2 Ui/Kg/day in the control group, meaning an increase of 27.6% from the baseline dose in the intervention group compared to an increase of 5.1% in the control group (Table 2 and Table 3). In addition, in eight (13.5%) subjects, the SMS insulin titration service reduced the basal insulin dose to prevent hypoglycemia. In the intervention group, we observed 12 (20.3%) subjects with poor adherence to SMS insulin titration. The main reasons were a lack of interest (eight subjects, 66.7%) and a lack of technological skills for interacting with the SMS service (four subjects, 33.4%).

**Figure 1:**
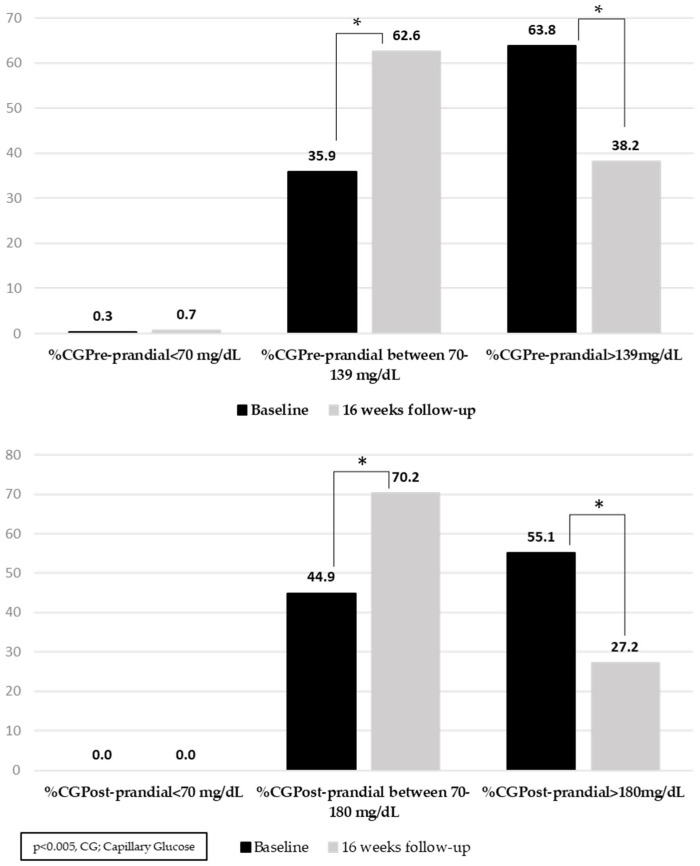
Percentage of capillary blood glucose within the target range. * *p* < 0.05.

The percentages of preprandial and postprandial capillary blood glucose measurements in the target in the interventional group are shown in Figure 1.

Regarding neuropsychological parameters, we observed changes from baseline to 16 weeks of follow-up in terms of several psychological aspects (Table 4).

The intervention resulted in a significant decrease in the proportion of participants with moderate/severe stress in the following DDS subscales: “Emotional Burden” (*p* = 0.03), “Interpersonal Distress” (*p* = 0.03) and, mainly, “Regimen Distress” (*p* < 0.001). This last datum indicates a very significant reduction in the stress associated with insulin treatment due to the use of the SMS titration guidance system.

In the HADS scale, we observed a significant decrease in the percentage of patients with Depression (*p* = 0.049). Finally, an improvement in mental health was detected in the SF-12 questionnaire.

To evaluate the participants’ satisfaction with the use of the SMS-based insulin titration service, we developed a questionnaire including seven specific questions (Table 5). The intervention was feasible, and patients were highly satisfied with their treatment.

The baseline differences between the poor adherence subjects and the subjects with an appropriate use of the SMS insulin titration service are detailed in Table 6. No extra contacts with the study staff concerning the use of SMS were reported. The multivariate analysis revealed that fewer years of education was an independent predictor of poor adherence to the SMS service (Table 7).

## 4. Discussion

The present study shows how an SMS-guided titration service was effective in improving glucometric parameters in subjects with T2D under basal insulin treatment, thus reducing the therapeutic inertia. Furthermore, an improvement in several psychological aspects was also observed.

The initiation of insulin therapy in T2D can be relatively straightforward, but the need for a timely follow-up to assure an optimal titration is a challenge, not only for subjects with diabetes but also for healthcare professionals and healthcare systems. Diabetes management based on telemedicine has already been proven to be effective in diabetes care [22,23,24,25,26,27]. At present, this strategy can be integrated into the current clinical practice worldwide, since most adults own a mobile phone and use SMS communication in their daily lives.

In our study, the percentage of subjects who achieved the target of FCBG at 16 weeks using the SMS insulin titration service was significantly higher than that of subjects under the standard of care. In addition, we also found significantly lower levels of HbA1c in the intervention group than in the control group, with a mean reduction of 0.4%. This reduction was similar to those obtained with several antidiabetic agents [28,29,30], but without any adverse effect. Furthermore, the mean time needed to achieve the FCBG on target was 5.1 weeks, and at this point, the SMS-driven titration service was finished. Therefore, a further reduction in HbA1c would be expected if the system was extended to the entire follow-up. A recent meta-analysis in which E-health interventions including SMS, smartphone applications and phone calls were compared showed that SMS was a lower-cost and lower-barrier technology in achieving the best effect in reducing HbA1c, with an optimal duration of ≤ 6 months [31].

The SMS-driven titration service improved other glucometric parameters such as preprandial and 2 h postprandial capillary blood glucose. In addition, it should be noted that all these improvements were achieved in the absence of severe hypoglycemic events and without increasing the number of mild hypoglycemic events from the baseline.

Few studies using an SMS service to improve basal insulin titration have been previously reported for subjects with T2D [22,23,24]. Kim et al. [22] demonstrated a higher reduction in HbA1c than in the present study, but it is important to consider that the subjects started from higher baseline glycosylated hemoglobin (9.8% vs. 8.3%). Langford et al. [24] reported that people with higher baseline fasting blood glucose levels had a lower chance of reaching optimal basal glycemic targets using an SMS service. Moreover, age, copay status and baseline fasting blood glucose were significantly associated with text message response rates.

Another key outcome of this analysis was the positive impact on psychological aspects. In fact, this is the first study showing an improvement in psychological parameters using a titration SMS-guided service. It is worth highlighting the stronger improvement in the stress associated with the insulin regimen, which can be very relevant in reducing therapeutic inertia. In addition, the participants were satisfied with the system and found it to be easy and friendly, thus reinforcing their self-confidence. Furthermore, it should be noted that the system is accessible and economic for the healthcare system.

Twelve subjects (20.3%) showed poor adherence to the SMS service. Several clinical and demographic statistical differences were observed in these participants, such as few years of education, a higher BMI, a higher prevalence of hypertension and dyslipidemia and more complications related to diabetes. Years of education was the only variable independently associated with poor adherence. This result could be useful for selecting people who are to benefit the most from the use of an SMS titration service. Since technology skills are associated with the level of education, more problems with regard to technology usage could occur in people with lower education levels [32].

This study also presents several limitations such as the sample size and the fact that it is not a randomized clinical trial (RCT). Thus, only the participants in the interventional arm performed the questionnaires, not being available for the standard-of-care group. Furthermore, a potential bias due to a higher level of concern regarding insulin treatment among subjects who agreed to participate in the interventional arm can exist. Nevertheless, the reduction in HbA1c observed in our study (0.40%) was similar to that reported in a previous meta-analysis (0.38%) based on 9 RCTs involving SMS [33]. It should be noted that these RCTs were conducted to improve medication adherence and not to optimize the insulin titration.

In conclusion, the SMS-guided titration service evaluated in the present study was effective in terms of improving glucometric parameters in comparison with the standard-of-care treatment. In addition, their use is easy and friendly and has a beneficial impact on several psychological aspects. Overall, this system may be an effective, easy-to-use tool in reducing the delay in insulin titration in our healthcare system.

## Figures and Tables

**Table 1:** Baseline characteristics of subjects with type 2 diabetes included in the study.

	Control Group	Intervention Group	*p*
n	59	59	
Age (years)	63.6 ± 11.7	64.1 ± 10.1	0.788
Sex (women) %	26 (44.1%)	26 (44.1%)	0.265
Smokers (n, %)			0.071
− Current (%)	6 (10.2%)	4 (6.8%)	
− Former (%)	10 (16.9%)	11 (18.6%)	
− Never (%)	43 (72.9%)	44 (74.6%)	
Body mass index (BMI) (kg/m^2^)	32.2 ± 4.6	31.4 ± 7.6	0.531
Hypertension (HTA) (%)	41 (69.5%)	40 (67.8%)	0.315
Dyslipidemia (DLP) (%)	42 (74.1%)	43 (74.1%)	0.258
T2D duration (years)	10.7 ± 5.4	11.5 ± 6.7	0.474
Time between T2D diagnosis and insulinization (years)	3.5 ± 2.5	3.5 ± 2.6	0.875
HbA1c (%) (mmol/mol)	8.3 ± 0.4	8.3 ± 0.8	0.952
	67 ± 3.2	67 ± 6.5	
Severe hypoglycemia (<54 mg/dL) (n)	0	0	n.s.
Mean FCBG at last week (mg/dL)	168.3 ± 24.9	167.9 ± 27.4	0.148
Insulin dose (UI/day)	30.4 ± 7.1	30.7 ± 11.5	0.354
Insulin dose (UI/Kg/day)	0.37 ± 0.20	0.38 ± 0.13	0.167
Fasting venous glucose (mg/dL)	169.5 ± 65.5	170.5 ± 57.5	0.101
Diabetes-related complications			
− Retinopathy (%)	9 (15.3%)	8 (13.6%)	0.194
− Nephropathy (%)	13 (22.0%)	12 (20.3%)	0.166
− Polyneuropathy (%)	8 (13.6%)	7 (11.9%)	0.319
− Ischemic heart disease (%)	5 (8.5%)	4 (6.8%)	0.752
− Stroke (%)	1	0	0.569
− Peripheral arteriopathy (%)	8 (13.6%)	7 (11.9%)	0.319

Data are expressed as the mean ± SD or percentage. Type 2 Diabetes (T2D).

**Table 2:** Evolution of glucometric parameters in the interventional group with appropriate adherence. Poor adherence was defined as reaching <80% compliance for the SMS response during follow-up.

	Baseline	16 Weeks of Follow-Up	*p*
n	47	47	
HbA1c (%) (mmol/mol)	8.3 ± 0.4	7.63 ± 0.1	<0.001
	67.0 ± 3.2	59.0 ± 0.8	
Fasting venous glucose (mg/dL)	166.5 ± 9.5	131.3 ± 5.9	<0.001
Last week mean fasting capillary blood glucose (mg/dL)	163.8 ± 3.5	123.4 ± 5.2	<0.001
Mean capillary blood glucose preprandial (mg/dL)	152.2 ± 5.1	130.3 ± 6.2	<0.001
Mean capillary blood glucose 2 h postprandial (mg/dL)	188.1 ± 6.9	160.8 ± 6.2	<0.001
Episodes of mild/moderate hypoglycemia (55–70 mg/dL) (n, %)	8 (17.0%)	4 (8.5%)	0.184
Episodes of severe hypoglycemia (<54 mg/dL) (n)	0	0	n.s
Insulin dose (UI)	28.3 ± 1.5	36.1 ± 3.2	<0.001
Insulin dose (UI/Kg/day)	0.35 ± 0.01	0.45 ± 0.04	<0.001
Fasting venous glucose (mg/dL)	166.5 ± 9.5	131.3 ± 5.9	<0.001

Data are expressed as the mean ± SD. Glycosylated hemoglobin (HbA1c).

**Table 3:** Evolution of glucometric parameters in the control group.

	Baseline	16 Weeks of Follow-Up	*p*
n	59	59	
HbA1c (%) (mmol/mol)	8.3 ± 0.2	7.9 ± 0.1	0.008
	67.0 ± 1.6	63.1 ± 0.8	
Fasting venous glucose (mg/dL)	165.1 ± 7.3	151.2 ± 3.4	0.011
Last week mean fasting capillary blood glucose (mg/dL)	168.3 ± 24.9	149.2 ± 6.0	0.021
Episodes of severe hypoglycemia (<54 mg/dL) (n)	0	0	n.s
Insulin dose (UI)	30.4 ± 7.1	31.9 ± 1.6	0.519
Insulin dose (UI/Kg/day)	0.37 ± 0.20	0.39 ± 0.2	0.519

Data are expressed as the mean ± SD. Glycosylated hemoglobin (HbA1c).

**Table 4:** Evolution of neuropsychological aspects.

Questionnaires	Baseline	16 Weeks of Follow-Up	*p*
n	47	47	
**DDS**			
Total Score (absolute)	2.1 ± 0.2	1.9 ± 0.2	0.082
Total Score (%)	48.5	34.4	0.21
Emotional Burden (absolute)	2.2 ± 0.2	1.9 ± 0.2	0.043
Emotional Burden (%)	47.6	30.9	0.03
Physician Distress (absolute)	1.8 ± 0.2	1.7 ± 0.2	0.529
Physician Distress (%)	30.6	25.0	0.57
Regimen Distress (absolute)	2.4 ± 0.2	1.4 ± 0.1	<0.001
Regimen Distress (%)	52.2	17.2	<0.001
Interpersonal Distress (absolute)	1.9 ± 0.2	1.4 ± 0.2	0.040
Interpersonal Distress (%)	27.0	16.2	0.03
**HADS**			
Anxiety (absolute)	11.7 ± 0.7	12.2 ± 0.5	0.347
Anxiety (%)	61.7	57.4	0.361
Depression (absolute)	9.4 ± 0.3	8.8 ± 0.3	0.179
Depression (%)	17.7	6.6	0.049
**SF-12**			
Physical (%)	47.9	44.1	0.091
Mental (%)	76.1	35.6	0.001
General (%)	65.8	50.8	0.02

Data are expressed as the mean ± SD of score values and as the % of participants with an abnormal score. Diabetes Distress Scale (DDS); Hospital Anxiety and Depression Scale (HADS); Social Functioning (SF-12).

**Table 5:** Results of the satisfaction questionnaire from patients that completed the study (n = 51).

	Not At All (%)			A Little Bit (%)	Neither Too Much Nor Too Little (%)		Considerable (%)		A Lot (%)	
**Do you think that (SMS) has been useful for your glycemic control?**										
Appropriate use	4.7			9.3	7.0		27.9		51.2	
Poor adherence subject	75			25	0		0		0	
**Are you satisfied with the (SMS) received?**										
Appropriate use	4.7			7.0	4.7		34.9		48.8	
Poor adherence subject	25			75	0		0		0	
**Would you like to continue receiving the SMS service?**										
Appropriate use	9.5			11.9	4.8		42.9		31.0	
Poor adherence subject	50			50	0		0		0	
**Was the Use of the SMS insulin titration service easy for you?**										
Appropriate use	2.3			9.3	7.0		39.5		41.9	
Poor adherence subject										
	**0 event (%)**			**1 event (%)**	**2–3 events (%)**		**3–5 events (%)**		**>5 events (%)**	
**Did you have mild/moderate hypoglycemia during the time you used the SMS insulin titration service?**										
Appropriate use	97.1			2.9	0		0		0	
Poor adherence subject	50			50	0		0		0	
**Did you have severe hypoglycemia during the time you used the SMS insulin titration service?**										
Appropriate use	100			0	0		0		0	
Poor adherence subject	100			0	0		0		0	
	**Nothing (%)**			**Sometimes (%)**			**Often (%)**		**A lot (%)**	
**Did the SMS insulin titration service bother you because it interrupted your daily routine?**										
Appropriate use	81.4			11.6			4.7		2.3	
Poor adherence subject	0			50			25		25	
	**1 (%)**	**2 (%)**	**3 (%)**	**4 (%)**	**5 (%)**	**6 (%)**	**7 (%)**	**8 (%)**	**9 (%)**	**10 (%)**
**How would you rate the usefulness of the SMS insulin titration service?** **(1—not at all useful and 10—very useful)**										
Appropriate use	0	2.1	0	0	2.1	2.1	2.1	14.9	27.7	48.9
Poor adherence subject	25	0	25	25	25	0	0	0	0	0

**Table 6:** Differences in the baseline characteristics of poor adherence subjects.

	Appropriate Use	Poor Adherence Subject	*p*
n	47	12	
Age (years)	63.5 ± 8.9	61.3 ± 14.1	0.503
Sex (women) %	19 (40.4%)	7 (58.3%)	0.265
Smoking			0.361
− Current (%)	4 (8.5%)	0	
− Former (%)	8 (17.0%)	3 (25.0%)	
− Never (%)	35 (74.5%)	9 (75.0%)	
Body mass index (BMI) (kg/m^2^)	30.2 ± 5.4	36.3 ± 12.5	0.013
Hypertension (HTA) (%)	35 (74.5%)	9 (75.0%)	0.336
Dyslipidemia (DLP) (%)	39 (82.9%)	9 (75.0%)	0.078
Education (years)	10.2 ± 3.4	5.1 ± 1.2	0.001
T2D duration (years)	11.7 ± 7.3	10.8 ± 3.4	0.653
Time between T2D diagnosis and insulinization (years)	3.5 ± 2.5	3.7 ± 2.7	0.875
HbA1c (%) (mmol/mol)	8.3 ± 0.8	8.3 ± 0.8	0.976
	67.1 ± 6.5	67.2 ± 6.5	
Mild/moderate hypoglycemia (55–70 mg/dL)	7 (14.9%)	1 (8.3%)	0.011
Severe hypoglycemia (<54 mg/dL) (n)	0	0	n.s
Last week mean fasting capillary blood glucose (mg/dL)	165.3 ± 24.9	178.1 ± 34.8	0.148
Basal insulin dose (UI/day)	28.5 ± 10.9	39.2 ± 10.3	0.004
Basal insulin dose (UI/Kg/day)	0.35 ± 0.12	0.36 ± 0.24	0.785
Fasting venous glucose (mg/dL)	164.4 ± 53.3	199.1 ± 70.3	0.101
Retinopathy (%)	5 (10.6%)	3 (25.0%)	0.004
Nephropathy (%)	9 (19.2%)	3 (25.0%)	0.036
Polyneuropathy (%)	3 (6.4%)	4 (33.3%)	0.010
Ischemic heart disease (%)	4 (8.5%)	0	0.295
Stroke (%)	0	0	n.s
Peripheral arteriopathy (%)	4 (8.5%)	3 (25.0%)	0.002

Data are expressed as the mean ± SD.

**Table 7:** Multivariate analysis for poor adherence to the SMS service.

	Confident	Standard Error	z	*p*	95% Conf. Interval	
Education (years)	−1.80165	0.63262	−2.85	0.004	−3.04156	−0.561746
Age (years)	0.00199	0.08591	0.02	0.982	−0.16638	0.17037
Body mass index (kg/m^2^)	0.94142	0.20615	0.46	0.648	−0.30991	0.49819
Diabetes-related complications (no/yes)	0.49433	2.98491	0.17	0.868	−5.35598	6.34464
_constant	9.35905	8.95606	1.04	0.296	−8.19452	26.9126

## Data Availability

The data are available from the corresponding authors upon request.

## Supplementary Materials

The following supporting information can be downloaded at: https://www.mdpi.com/article/10.3390/jcm12196364/s1, Table S1. Study schedule.
