# Supplementary material for: Efficacy of Insulin Titration Driven by SMS in Improving Glycemic Control in People with Type 2 Diabetes
Source: J Clin Med. 2023 Oct 4;12(19):6364. doi: 10.3390/jcm12196364 (PMC10573537; doi:10.3390/jcm12196364)
Supplement: Supplementary file 1 [file jcm-12-06364-s001.zip › jcm-2588636-supplementary.pdf]

Journal of Clinical Medicine

Supplementary Table S1. Study schedule

	**Screening visit**	**Baseline visit Visit 1**	**Virtual Visit Visit 2**	**Final Visit Visit 3**
	Day-21-Day -7	Day 1	*	Week 16-18
Informed consent	X			
Confirm Inclusion/Exclusion criteria	X			
Medical history	X	X		X
Hypoglycemic events		X		X
Insulin and concomitant Medication	X	X		X
FCBG measurements [a]	X	X		X
Post-prandial CBG [b]		X		X
HbA1C	X	X		X
Training on a SMS based insulin titration service		X		
Adherence to the requests of a SMS based insulin titration service				X
Questionnaires [c]		X		X
Subject satisfaction regarding the use of a SMS based insulin titration service				X
HCP satisfaction regarding the use of a SMS based insulin titration service				X

FCBG: fasting capillary blood glucose

[a] At least 3 days in the last week

[b] A complete profile (2 hours post breakfast, lunch, and dinner) 2 days a week.

[c] DDS, HADS, SF12. All these questionnaires are translated to Spanish.

* Time point when the target FCBG is achieved
